# Identification of the flavivirus conserved residues in the envelope protein hinge region for the rational design of a candidate West Nile live-attenuated vaccine

**DOI:** 10.1038/s41541-023-00765-0

**Published:** 2023-11-06

**Authors:** Bailey E. Maloney, Kassandra L. Carpio, Ashley N. Bilyeu, Danielle R. D. Saunders, So Lee Park, Adrienne E. Pohl, Natalia Costa Ball, Janae L. Raetz, Claire Y. Huang, Stephen Higgs, Alan D. T. Barrett, Gleyder Roman-Sosa, Joanie L. Kenney, Dana L. Vanlandingham, Yan-Jang S. Huang

**Affiliations:** 1 Department of Diagnostic Medicine/Pathobiology, College of Veterinary Medicine, Kansas State University, Manhattan, KS 66506 USA; 2 Biosecurity Research Institute, Kansas State University, Manhattan, KS 66506 USA; 3 Department of Biochemistry and Molecular Biology, University of Texas Medical Branch, Galveston, TX 77555 USA; 4 Sealy Institute for Vaccine Sciences, University of Texas Medical Branch, Galveston, TX 77555 USA; 5 Division of Vector-borne Diseases, Centers for Disease Control and Prevention, Fort Collins, CO 80521 USA; 6 Department of Pathology, University of Texas Medical Branch, Galveston, TX 77555 USA; 7 Present Address: Department of Biology, Dean of Faculty, United States Air Force Academy, Colorado Springs, CO 80840 USA; 8 Present Address: Institute of Virology, University of Veterinary Medicine Hanover, Foundation, Buentewg 17, 30559 Hanover, Germany; 9 Present Address: Department of Microbiology and Immunology and SUNY Center for Vector-Borne Diseases, Institute of Global Health and Translation Science, Upstate Medical University, Syracuse, NY 13210 USA

**Keywords:** Viral membrane fusion, West nile virus, Live attenuated vaccines

## Abstract

The flavivirus envelope protein is a class II fusion protein that drives flavivirus-cell membrane fusion. The membrane fusion process is triggered by the conformational change of the E protein from dimer in the virion to trimer, which involves the rearrangement of three domains, EDI, EDII, and EDIII. The movement between EDI and EDII initiates the formation of the E protein trimer. The EDI-EDII hinge region utilizes four motifs to exert the hinge effect at the interdomain region and is crucial for the membrane fusion activity of the E protein. Using West Nile virus (WNV) NY99 strain derived from an infectious clone, we investigated the role of eight flavivirus-conserved hydrophobic residues in the EDI-EDII hinge region in the conformational change of E protein from dimer to trimer and viral entry. Single mutations of the E-A54, E-I130, E-I135, E-I196, and E-Y201 residues affected infectivity. Importantly, the E-A54I and E-Y201P mutations fully attenuated the mouse neuroinvasive phenotype of WNV. The results suggest that multiple flavivirus-conserved hydrophobic residues in the EDI-EDII hinge region play a critical role in the structure–function of the E protein and some contribute to the virulence phenotype of flaviviruses as demonstrated by the attenuation of the mouse neuroinvasive phenotype of WNV. Thus, as a proof of concept, residues in the EDI-EDII hinge region are proposed targets to engineer attenuating mutations for inclusion in the rational design of candidate live-attenuated flavivirus vaccines.

## Introduction

The *Flavivirus* genus contains arthropod-borne viruses of major public health importance, including dengue (DENV), West Nile (WNV), and Zika (ZIKV) viruses^1^. Flaviviruses cause millions of human infections each year and could lead to life-threatening encephalitic, viscerotropic, and/or hemorrhagic diseases. No licensed antiviral therapy exists for any flavivirus. There are vaccines for five flaviviruses but effective live-attenuated vaccines (LAVs) that elicit protective immunity with one single immunization are only available for Japanese encephalitis (JE) and yellow fever (YF) viruses. Other licensed flavivirus vaccines require multiple doses to achieve immune protection, including the three-dose dengue (DENV) LAV, and two-dose inactivated vaccines for JE, tick-borne encephalitis (TBE), and Kyasanur Forest Disease. As the empirical attenuation approach that developed JE and YF LAVs is not appropriate for most flaviviruses, there is a growing interest in the rational design of candidate LAVs.

The rational design of candidate LAVs requires mutations to reach the balance between attenuation and immunogenicity^2,3^. The flavivirus genome consists of a single-stranded positive-sense RNA that contains a single open reading frame that encodes three structural [capsid, pre-membrane, and envelope (E)] and seven nonstructural (NS1, NS2A, NS2B, NS3, NS4A, NS4B, and NS5) proteins. All flaviviruses are evolutionarily related. The motifs of consensus sequences in viral-encoded proteins are often the targets for the engineering of flavivirus-common attenuating mutations to support the rational design of candidate LAVs^2,4–12^. The E protein is a major structural protein in the virion and exhibits up to 40% amino acid sequence homology between flaviviruses. Surprisingly, to date, few mutations have been identified in the E protein that can attenuate the virulence phenotype of a broad range of flaviviruses^13–16^. The flavivirus E protein is a class II fusion protein and the conformational change of the E protein from dimer in the virion to trimer drives the membrane fusion process^17,18^. The fusion of viral and cell membranes releases the viral genome into the cytoplasm and is the conserved mechanism of viral entry^19^. Therefore, we propose that knowledge of the flavivirus conserved motifs controlling the conformational change of the E protein from dimer in the virion to trimer can be translated to support the development of a flavivirus-common attenuation strategy.

The E protein is found as a dimer in virions and each monomer consists of three distinct domains, EDI, EDII, and EDIII^20^. The shift of EDII away from the virion surface against EDI causes the dissociation of the E protein dimer^17^. Subsequently, three copies of the E protein monomer form the trimeric structure that exhibit the membrane fusion activity. The four motifs at the interface of EDI and EDII exert the hinge effect critical for the movement of EDII, hence the name EDI-EDII hinge region. Sequence alignment of the EDI-EDII hinge region of different flaviviruses identifies a consensus sequence that contains eight flavivirus-conserved hydrophobic amino acids, several of which have the implicated importance for membrane fusion process and the virulence phenotype of flaviviruses (Table 1)^3,20–30^. Since there are eight flavivirus-conserved residues the EDI-EDII hinge region, including those previously shown to be critical for the conserved structure–function of the DENV-2 E protein^31^, we hypothesized that mutation of one or more of these eight residues would attenuate the virulence phenotype of flaviviruses.

**Table 1:**
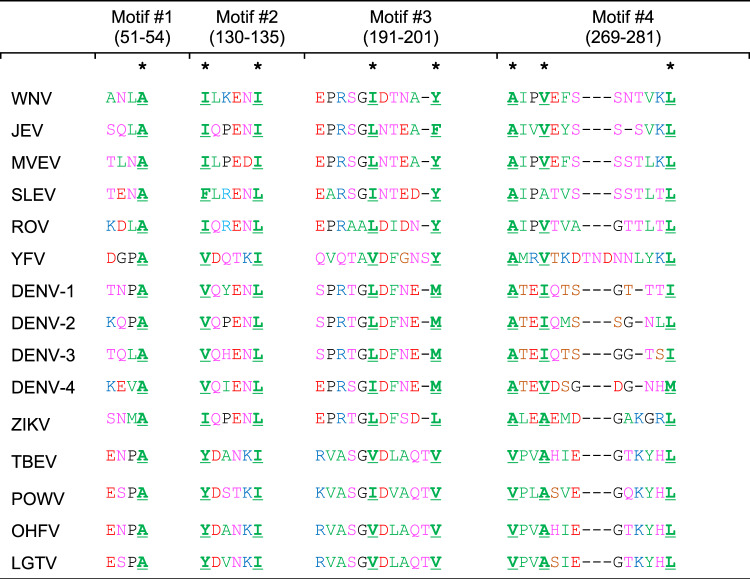
Amino acid sequences of the EDI-EDII hinge region among flaviviruses.

The properties of amino acids are shown in green (hydrophobic), pink (polar), red (negatively charged), blue (positively charged), and black (unique). The numbers correspond to the amino acid positions in WNV E protein. The asterisk symbol is used to highlight the eight conserved hydrophobic residues in the four different motifs of EDI-EDII hinge region. Genbank access numbers: WNV-AF253491, JEV-M18370, MVEV-X03467-, St. Louis encephalitis virus (SLEV)-AY289618, Rocio virus (ROV)- MF461639, YFV-AY640589, DENV-1-KM204119, DENV-2-AF038403, DENV-3-M93130, DENV-4-AY947539, ZIKV-MW143022, tick-borne encephalitis virus (TBEV)-U27495, Powassan virus (POWV)- L06436, Langat virus (LGTV)-AF253419, Omsk hemorrhagic fever virus (OHFV)- AB507800.

In this study, we used a cDNA infectious clone of WNV NY99 strain (WNV NY99ic) to determine the attenuating effect caused by single mutations of the eight flavivirus-conserved hydrophobic amino acids in the EDI-EDII hinge region on the mouse neuroinvasive phenotype^32^. Our hypothesis is that mutations of the conserved hydrophobic residues in the EDI-EDII hinge region will interfere with cell entry and consequently attenuate the mouse neuroinvasive phenotype of WNV. Single amino acid substitutions were engineered to replace each conserved hydrophobic residue in the EDI-EDII hinge region with an amino acid of lower hydrophobicity, and we identified mutations of the E-A54 and E-Y201 residues that can interfere with the cell entry of WNV, reduce the multiplication kinetics of WNV in Vero cells, and attenuate the mouse neuroinvasive phenotype. Thus, the results from cell culture and mouse experiments demonstrate that multiple flavivirus conserved hydrophobic amino acids in the EDI-EDII hinge region are important for the structure–function of WNV E protein and the neuroinvasive phenotype of WNV in mice. They provide a proof-of-concept that rational design of a flavivirus live-attenuated vaccine could include mutations of residues in the E protein EDI-EDII hinge region.

## Results

### Recovery of WNV mutants

Each of the eight flavivirus-conserved residues in the EDI-EDII hinge region was mutated as follows: A54S, I130N, I135N, I196N, Y201P, A269S, V272T, and L281N and rescue of the mutants was attempted following transfection of Vero cells. Six of the mutants exhibited phenotypes indicative of disruption of the structure–function of the E protein, including reduced infectivity, lethal phenotype, and reversion to the consensus wt NY99ic sequence.

The single E-I130N, E-I135N, and E-I196N mutations were lethal to WNV NY99ic. In two of three biological replications of transfection experiments, the E-I196N mutation reverted to the wt sequence. The lack of detectable infectivity suggests that replication of RNA transcripts likely occurred at a low level, led to the reversion but failed to produce infectious mutant viruses. Collectively, the E-I130, E-I135 and E-I196 residues are crucial for the structure–function of the E protein.

In contrast, the E-A269S and E-V272T mutants each had the correct mutation but resembled the multiplication kinetics of WNV NY99ic, exceeding 8 log_10_ pfu/ml. The E-L281N mutation involved the change of all three nucleotides in the codon, but it still reverted to the wt sequence in two independent transfections, suggesting that the E-L281 residue is very important for the structure–function of the E protein.

**Fig. 1:**
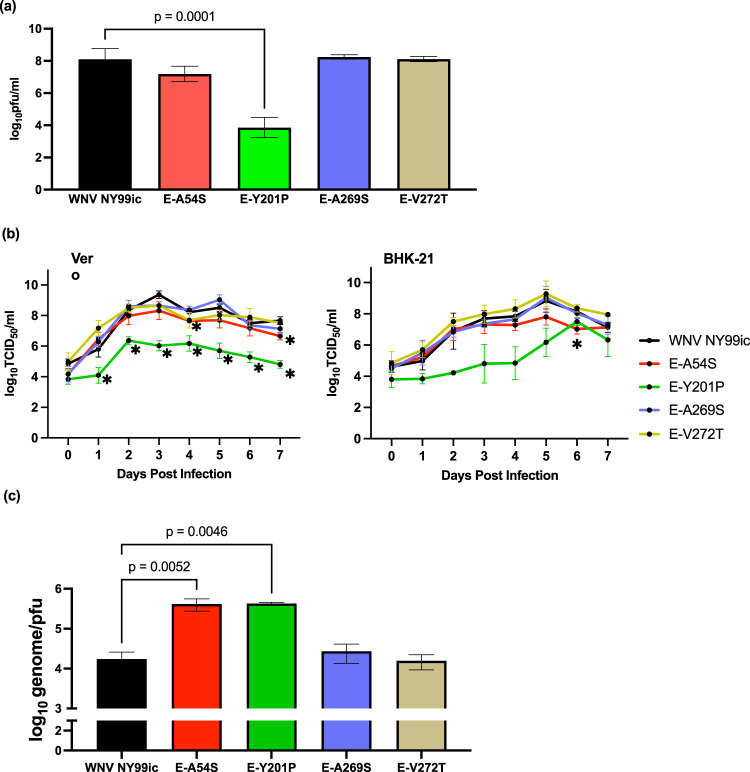
The single E-A54S and E-Y201P mutations reduce the infectivity of WNV NY99ic in Vero cells. **a** Infectivity of WNV NY99ic and the four single mutants, following transfection of Vero cells. Infectivity between WNV NY99ic and each mutant was compared using one-way ANOVA followed by Dunette’s pairwise comparison. **b** Multiplication kinetics of WNV NY99ic and the four single mutants in Vero cells and BHK-21 cells infected at MOI of 0.1. Data points represent the mean viral titer and error bars represent the standard deviation of three biological replicates. The asterisk sign indicates the significant difference (*P* < 0.05) detected by two-way ANOVA followed by Dunett’s pairwise comparison at a specific timepoint. **c** Genome-pfu ratios of WNV NY99ic and the four mutants in the culture supernatants of Vero cells infected at MOI of 0.1. Data points represent the average genome copy-to-pfu ratios and error bars represent the standard deviation of two biological replicates prepared from Vero cells infected with WNV NY99ic or each mutant at MOI of 0.1. Comparison of genome copy-to-pfu ratio was performed using one-way ANOVA followed by Dunnett’s pairwise comparison.

The E-Y201P mutation on the third motif of the EDI-EDII hinge region reduced the infectivity of WNV NY99ic by 5400-fold (*P* = 0.0001) (Fig. 1a). The average titer of the E-A54S mutant was 2.5-fold lower than WNV NY99ic, although there was a lack of statistical significance (*P* = 0.43). Both the E-Y201P and E-A54S mutations were retained in the consensus sequence determined by Sanger sequencing.

### Reduced multiplication kinetics of WNV E-A54S and E-Y201 mutants in Vero and BHK-21 cells

The multiplication kinetics of four mutants that retained the amino acid substitutions (E-A54S, E-Y201P, E-A269S, and E-V272T) were determined in Vero and BHK-21 cells (Fig. 1b). The E-A54S and E-Y201P mutants exhibited reduced infectivity compared to NY99ic, resembling the attenuated WNV mutants previously reported^7,9,33^. The multiplication kinetics of the E-Y201P mutant in Vero cells was reduced by up to 1000-fold in comparison to WNV NY99ic between 1 and 7 dpi (0.0005 ≤ *P* ≤ 0.047). While the infectivity of the E-Y201P mutant did not differ significantly from WNV NY99ic in BHK-21 cells (0.08 ≤ *P* ≤ 0.65), the general trend showed the reduced infectivity of the E-Y201P by over 13-fold between 2 and 5 dpi. The multiplication kinetics of the E-A54S mutant and WNV NY99ic exhibited no demonstrable differences in both Vero and BHK-21 cells prior to 3 dpi, but the infectivity of the E-A54S mutant differed from WNV NY99ic in Vero cells at the plateau phase, reaching statistical significance at 4 (3.7-fold, *P* = 0.0001) and 7 (tenfold, *P* = 0.02) dpi. Similarly, the infectivity of the E-A54S mutant was approximately 13-fold lower than WNV NY99ic in BHK-21 cells, leading to the significant difference detected at 6 dpi (*P* = 0.04). The E-A269S and E-V272T mutants resembled the multiplication kinetics of WNV NY99ic, exceeding 8 log_10_TCID_50_/ml in Vero cells between 3 and 7 dpi and BHK-21 cells between 5 and 7 dpi.

### Single E-A54S and E-Y201P mutations can impair the infection of host cell with WNV

We determined the genome-pfu ratio of each mutant to investigate whether or not the reduced infectivity of the E-A54S and E-Y201P mutant is due to release of noninfectious particles in cell culture (Fig. 1c). The genome-pfu ratio of the E-A54S and E-Y201P mutants was 25-fold (*P* = 0.0052) and 28-fold (*P* = 0.0046) higher than WNV NY99ic, respectively. In contrast, neither the E-A269S mutation nor the E-V272T mutation led to demonstrable differences in the genome-pfu ratio in comparison with WNV NY99ic. Because the EDI-EDII interdomain movement initiates the formation of the E protein trimer, which in turn leads to the HA activity of flaviviruses, we examined the HA titer of each mutant at a range of pHs between pH 5.8 and pH 7.4. We hypothesized that differences in the optimal pH that leads to the highest HA activity will provide evidence that mutations of the flavivirus-conserved hydrophobic amino acids in the EDI-EDII hinge region can interfere with the conformational change of the E protein from dimer to trimer. WNV NY99ic exhibited the highest HA titer at pH 7.0–7.2, which was also the optimal pH for the HA activity of the E-A54S, E-A269S, and E-V272T mutants (Table 2). In comparison, the E-Y201P mutation reduced the optimal pH for the HA activity to pH 6.6 −6.8. Collectively, both the E-A54S and E-Y201P mutations interfere with the formation of infectious viruses, and the E-Y201 residue is crucial for the conformational change of the E protein from dimer to trimer as shown by alteration of the optimum pH for HA activity.

**Table 2:** Optimal pH for the HA activity of WNV NY99ic and each single mutant.

Virus	Optimal pH for HA activity
WNV NY99ic	7.0–7.2
E-A54S	7.2
E-Y201P	6.6–6.8
E-A269S	6.8–7.2
E-V272T	7.0
E-L281N	6.8–7.2

Geometric mean HA titer of WNV NY99ic and each mutant were determined by two biological replicates of HA test at gradient pH between pH 5.8 and pH 7.4 using 0.5% gander erythrocytes.

### Genetic stability of the E-A54S and E-Y201P mutants on the passage in Vero cells

**Fig. 2:**
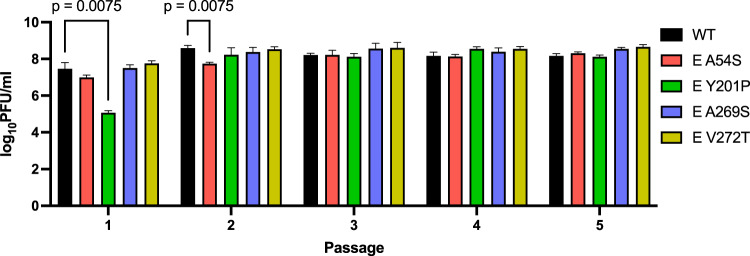
Serially passaged mutants demonstrated the E-A54S mutation is retained and tolerated by WNV NY99ic and the replacement of single-site E-Y201P mutation with leucine restored the infectivity of WNV NY99ic. Data points represent the average titers and error bars represent the standard deviation of three biological replicates prepared from Vero cells infected with WNV NY99ic or each mutant at MOI of 0.1. Infectivity at each passage was compared by two-way ANOVA and Dunnett’s pairwise comparison.

The E-A54S and E-Y201P mutants were given five passages in Vero cells at a MOI of 0.1. Infectivity of E-Y201P mutant became comparable to WNV NY99ic in the second passage, when the cytosine to uracil transition at the genome position 1568 changed the codon encoding proline to a leucine residue in the consensus sequence (Fig. 2) suggesting that the E-Y201 residue are crucial for the structure–function of the E protein. In contrast, the E-A54S mutation was retained after five passages despite the E-A54 residue is also important for the infectivity of WNV. Neither the E-A269S mutant nor the E-V272T mutant reverted to the wt sequence or accumulated compensatory mutations after five passages in Vero cells.

### Attenuation of the mouse neuroinvasive phenotype of WNV by E-A54S and E-Y201P mutants

**Fig. 3:**
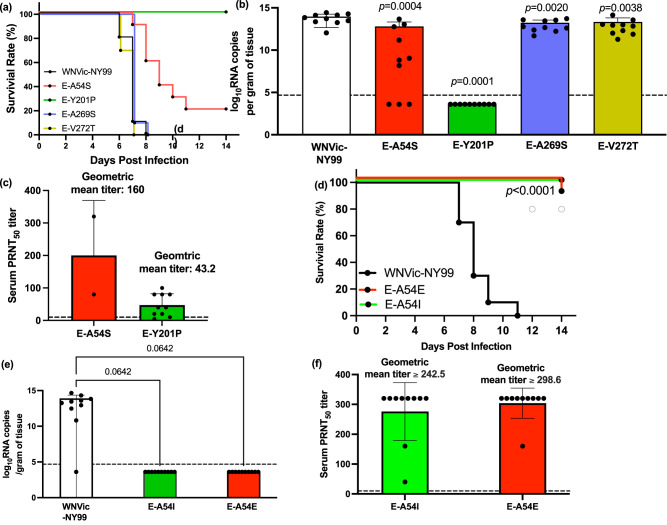
Mutations of the E-A54 and E-Y201 residues can attenuate the neuroinvasive phenotype of WNV NY99ic in 4-week-old mice. Groups of five male mice and five female mice received the i.p. challenge with each of the WNV mutants or WNV NY99ic at 500 pfu. **a** Survival rate of mice receiving the E-A54S, E-Y201P, E-A269S, E-V272T mutants or the parental WNV NY99ic. Difference in the survival outcomes and average survival time were shown by the Kaplan–Meier survival curve and one-way ANOVA followed by Dunnett’s pairwise comparison, respectively. **b** Brain viral load of mice challenged with WNV NY99ic or the respective single mutant was compared by one-way ANOVA and Dunnett’s pairwise comparison. Dash line indicates limit of detection. **c** Serum PRNT_50_ titer of mice surviving the challenge with the E-A54S or E-Y201P mutants. Dash line indicates 1:10 serum PRNT_50_ titer. **d** Survival rate of mice challenged with the E-A54I, E-A54E mutants or the parental WNV NY99ic. **e** Brain viral load of mice challenged with WNV NY99ic or the respective single E-A54 mutants. Dash line indicates limit of detection. **f** Serum PRNT_50_ titer of mice surviving the challenge with the E-A54I or E-A54E mutants. The geometric mean titers are shown in greater than or equal to because serum PRNT_50_ titers of seven mice surving the challenge with the E-A54I mutant and eight mice surviving the challenge with the E-A54E mutant did not reach the endpoint at 1:320 dilution. Dash line indicates 1:10 serum PRNT_50_ titer.

The neuroinvasive phenotype of each mutant was compared with WNV NY99ic in 4-week-old outbred Swiss mice following i.p. inoculation of 500 pfu (Fig. 3a). Infection with the wt WNV NY99ic led to uniform lethality between 6 and 8 dpi. Significantly, no mortality was observed in mice challenged with the E-Y201P mutant (*P* < 0.0001) and no viral RNA was detected in the brains (limit of detection = 4.8 × 10^4^ genome copies/gram of tissue) collected at day 14 pi (Fig. 3b). Therefore, the E-Y201P mutation fully attenuated the neuroinvasive phenotype in 4-week-old Swiss Webster mice challenged with 500 pfu. The E-A54S mutation exhibited a partially attenuated phenotype, as 20% of mice survived up to 14 dpi and had an extended average survival time of 9.8 days in comparison to that of WNV NY99ic at 6.9 days (*P* < 0.01). Mice challenged with the E-A54S mutant had 14-fold lower brain viral load (6.5 × 10^12^ genome copies/gram of tissue) than those challenged with WNV NY99ic (9.3 × 10^13^ genome copies/gram of tissue) (*P* = 0.001). And, two mice that survived the challenge with the E-A54S mutant had no detectable viral RNA in the brain at 14 dpi. Although the neuroinvasive phenotype of the E-A269S and E-V272T mutants was indistinguishable from WNV NY99ic and caused 100% mortality between 6 and 8 dpi, there was a significant reduction of viral load in the brain collected upon euthanasia (E-A269S mutant: 1.7 × 10^13^ genome copies/gram of tissue and fivefold reduction, *P* = 0.002; E-V272T mutant: 2.2 × 10^13^ genome copies/gram of tissue and fourfold reduction, *P* = 0.004).

Both the E-Y201P and E-A54S mutants elicited neutralizing antibody responses in surviving mice. Nine mice (90%, 9/10) that survived the i.p. challenge with the E-Y201P mutant showed 1:10 or greater serum PRNT_50_ titer (geometric mean of serum PRNT_50_ titer: 43.2) (Fig. 3c). Similarly, both mice that survived the i.p. challenge with the E-A54S mutant also had a detectable serum-neutralizing activity (geometric mean of serum PRNT_50_ titer: 160). Albeit the small sample size, the fully attenuated E-Y201P mutant exhibited lower immunogenicity than the partially attenuated E-A54S mutant.

Because the E-A54S mutation retains the small side chain and only leads to the partially attenuated phenotype, we investigated the attenuating effect of the E-A54I and E-A54E mutations, which contains a large hydrophobic and an acidic side chain, respectively. The E-A54I mutant was fully attenuated for the mouse neuroinvasive phenotype and the E-A54E mutant was highly attenuated, i.e., 10% mortality rate, at the inoculum of 500 pfu (Fig. 3d). Mice surviving the challenge with either the E-A54I or E-A54E mutants had no detectable viral RNA in the brain (Fig. 3e). Neither the E-A54I mutation nor the E-A54E mutation compromised the immunogenicity of WNV, as observed with the geometric mean of PRNT_50_ titers (E-A54I mutant: ≥242.5; E-A54E mutant: ≥298.6) (Fig. 3f).

### Next-generation sequencing (NGS) of WNV mutants

**Fig. 4:**
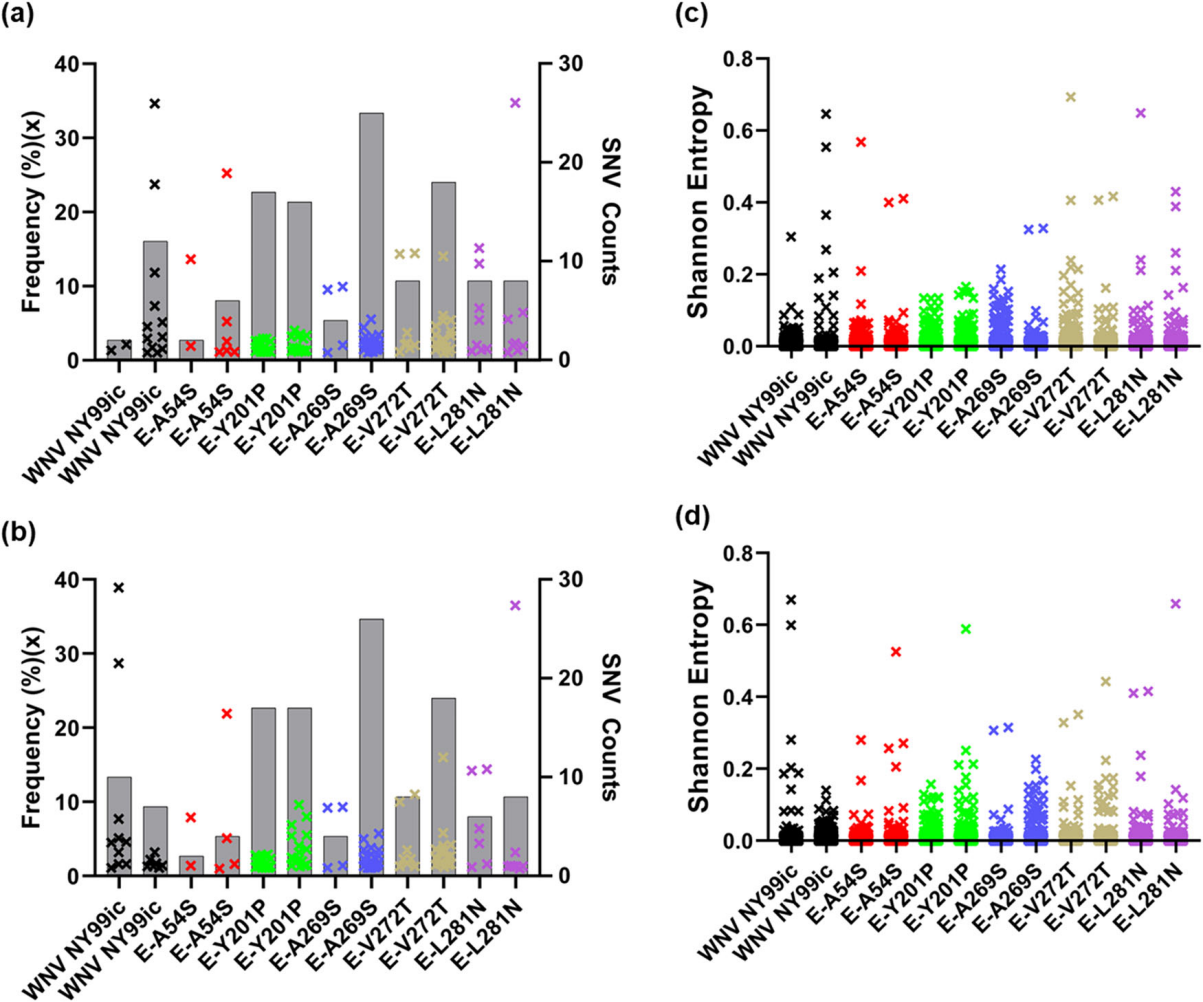
The E-A54S and E-Y201P mutants have RNA populations similar to WNV NY99ic. The SNV profiles of the two P0 stocks and two P1 stocks of each virus are summarized in (**a**, **b**), respectively. Each × represents a SNV above 1% of RNA population in the left *y* axis. Gray bars denote the SNV count of each virus in the right *y* axis. The Shannon entropy of the two P0 stocks and two P1 stocks of each virus is shown in (**c**, **d**), respectively. Each × indicates the nonzero Shannon entropy found within the genome of each virus.

To investigate the RNA population of the mutants we undertook the NGS of the P0 and P1 stocks of WNV NY99ic and the five viable single mutants, each of which contains the single polar amino acid substitution to replace the flavivirus-conserved E-A54, E-Y201, E-A269, E-V272, or E-L281 hydrophobic residues. Both NY99ic and each of the mutants were rescued twice on separate occasions to obtain confirmatory data. In contrast to the retention of the single E-A54S, E-Y201P, E-A269S, and E-V272T mutations in the consensus sequence in the P0 stocks, there was evidence of selection pressure within the E-L281N mutant viral RNA population. There was a strong selection for the E-L281N mutation to revert to the wt sequence in the P0 stock as evidenced by no detectable SNVs in the vRNA population encoding for the N residue (AAC) even though all three nucleotides in the codon need to mutate to wt NY99ic, i.e, AAC to GUU. The single nucleotide E-A54, E-Y201P, E-A269S, and E-V272T mutants have similar number or frequencies of SNVs to at least one of the P0 WNV NY99ic stocks (Fig. 4a and Supplementary Table 1). There were 12 SNVs ranging from 1.0 to 34.6% in the two biological replicates of WNV NY99ic, which is comparable to the 14 SNVs ranging from 1.0 to 34.7% in the E-L281N mutant that reverted to the wt sequence. The E-A54S mutant contained seven SNVs ranging from 1.0 to 25.2%, the E-Y201P mutant had 31 SNVs ranging from 1.1 to 4.0%, the E-A269S mutant had 28 SNVs ranging from 1.0 to 9.9%, and the E-V272T mutant had 24 SNVs ranging from 1.0 to 14.4%. None of the SNVs identified in the four mutants were detected at positions of nucleotides involved in the mutations. Most SNVs detected in the P0 stock were present in the passage one (P1) stocks of WNV NY99ic and the respective mutants (Fig. 4b and Supplementary Table 2). The sequence diversity of the respective mutants is similar to the WNV NY99ic, as observed with the Shannon entropy (Fig. 4c, d).

Neither of the attenuated mutants (E-Y201P and E-A54S mutants) contained SNVs indicative of reversion to the wt sequence or compensatory mutations in the P0 stocks used for the mouse neuroinvasive phenotype study. Further, we did not detect any SNV encoding reversion to the wt sequence or compensatory mutations in the P0 and P1 stocks of the fully attenuated E-A54I mutant (Supplementary Tables 3 and 4), generated to optimize the attenuating effect caused by amino acid substitutions of the E-A54 residue. Similarly, engineering of the E-A54E mutation did not lead to reversions or compensatory mutations (Supplementary Tables 3 and 4). Thus, the E-A54E mutant has similar genetic diversity to the E-A54I mutatant, so they would be expected to be equally as stable upon continued passaging.

The E-Y201P mutation was introduced by changing the nucleotides at positions 1567 and 1568. Interestingly, one of the two P1 stocks of the E-Y201P mutant contained two SNVs that lead to a nonsynonymous mutation, including the C1568U transition in 9.6% of reads and the C1568A transversion in 8.0% of reads. Following two passages in Vero cells, the C1568U transition that gave rise to a L residue rather than the C1568A transversion that encodes a histidine residue became a consensus mutation. Selection of the C1568U transition suggests that a hydrophobic amino acid in the E-Y201 residue is important for the structure–function of the E protein. Both the P0 and P1 stocks of the E-A269S mutant developed an SNV of the adenosine to cytosine transversion at the genome position 1774 present in >9% of reads, which encodes a E-I270L mutation in the vRNA population, but not the consensus sequence, at least at passages P0 and P1. The E-I270L mutation is next to the E-A269 residue, making it a compensatory mutation due to the structural and functional change caused by the E-A269S mutation.

## Discussion

In this study, we investigated the role of eight flavivirus hydrophobic residues in the EDI-EDII hinge region in the conformational change of WNV E protein from dimer to trimer. Since only one substitution was investigated for each flavivirus-conserved residue, except for E-A54, we can only limit our conclusions to those specific substitutions analyzed here. However, we believe that results present a proof-of-concept that mutations in the E protein EDI-EDII hinge region can attenuate at least WNV and that they have the potential to be included as attenuating mutations in a rationally designed LAV. We demonstrated that seven of the eight flavivirus-conserved hydrophobic amino acids in the EDI-EDII hinge region are important for the structure–function of WNV E protein, including the E-A54, E-I130, E-I135, E-I196, E-Y201, E-A269, and E-L281 residues. The importance of the E-I130, E-I196, E-Y201, E-A269, and E-L281 residues has not been previously reported. The single nucleotide E-I130N, E-I135N, and E-I196N mutants were not viable and the E-L281 residue was crucial for the structure–function of the E protein, as demonstrated by the reversion to the wt sequence in P0 stocks. Previously, the E-A54 and E-I135 residues have been shown to be important for the infectivity of DENV-2^31^ and so we have extended this observation to WNV, which is in a different genetic group of the Flavivirus genus. The E-A54S and E-Y201P mutations caused reduction in the infectivity of WNV. The increased genome copy-to-pfu ratios suggest that both the E-A54S and E-Y201P mutations can impair the establishment of WNV infection in the host cell. The reduced infectivity of WNV E-Y201P mutant can be attributed to the interference with the conformational change of the E protein from dimer in the virion to trimer and membrane fusion process based on the lower optimal pH for the HA activity, which would suggest that the WNV E-Y201P mutant requires more free energy provided by protonation of the endosome to trigger the conformational change from dimer to trimer.

We hypothesized that mutations of conserved hydrophobic amino acids in the EDI-EDII hinge region would attenuate the virulence phenotype of WNV in mice. We used the outbred Swiss Webster mice, which are highly susceptible to neuroinvasive disease following i.p. inoculation of WNV NY99ic^34^. The E-Y201P mutation fully attenuated the neuroinvasive phenotype of WNV at an inoculum of 500 pfu. It is worth noting that 500 pfu is equivalent to ~1000 50% lethal dose (LD_50_) of WNV NY99ic and can be considered a high dose for i.p. challenge^9^. While the attenuated phenotype of the E-A54S mutant was only partial, the E-A54I mutant that has a large hydrophobic side chain can fully attenuate the mouse neuroinvasive phenotype of WNV. We propose that mutations of the conserved hydrophobic residues in the flavivirus EI-EII hinge region offer a more effective attenuation strategy than the previously described mutations in the fusion loop, which can only partially attenuate the mouse neuroinvasive phenotype of WNV and were shown to revert to the wt sequence in the brains of mice succumbing to neuroinvasive disease^13^. In contrast, mice surviving i.p. challenge with either the E-A54S, E-A54I, E-A54E, or E-Y201P mutants had no detectable viral RNA in the brain indicating little potential of the mutants to revert to virulence and mice developed serum-neutralizing antibodies, suggesting the two attenuating mutations do not compromise the immunogenicity of candidate LAVs. Serum-neutralizing activity of mice surviving infection with either of the four mutants exceeds 1:10 geometric mean PRNT_50_ titer. Serum-neutralizing antibodies are the correlate of protection for candidate WN vaccines in mouse models^35^. Relevant to our studies, ICR mice that developed geometric mean PRNT_50_ titer of 37 or greater were fully protected against challenge with 1000 pfu of WNV NY99 strain, in comparison with partial protection of ICR mice that developed geometric mean PRNT_50_ titer of 20 (see ref. ^3^). Passive protection studies also showed that a serum PRNT_50_ titer of 45 conferred full protection of outbred ddY mice challenged with WNV NY99 strain at 6400 pfu^36^. Therefore, we expect that neutralizing antibodies raised against the E-A54 mutants and the E-Y201P mutant can potentially protect against a lethal dose of challenge up to 1000 pfu. We also do not anticipate that single amino acid substitutions in the E protein will compromise the presentation of B-cell and T-cell epitopes in other viral proteins, warranting investigation of immunogenicity of the fully attenuated E-A54I and E-Y201P mutants. Neither the E-A54I mutant nor the E-Y201P mutant developed reversion to the wt sequence or compensatory mutations in the consensus sequence of P0 and P1 stocks. We expect that manufacturing of candidate WN LAVs containing the E-A54I or E-Y201P mutatios will require a seed lot system monitored by NGS initially for SNVs encoding compensatory mutations in the RNA population. There have been multiple flavivirus LAVs manufactured using seed lot systems to prevent the reversion to the virulence phenotype, including YF 17D, JE SA14-14-2, IMOJEV® chimeric JE, and Dengvaxia® chimeric DEN LAVs^37–39^.

Due to the conservation of residues between flaviviruses in the EDI-EDII hinge region, we believe that mutations of the WNV E-A54 and E-Y201 residues have the potential to be translated to support the development of a flavivirus-common attenuation mechanism by affecting the conserved hydrophobic biochemical property critical for E protein function. The E-A54 residue is strictly conserved between all known flaviviruses, and the E-A54E mutation has been shown to interfere with the membrane fusion process of DENV-2^31^. The amino acid equivalent to the WNV E-Y201 residue also exhibits a hydrophobic biochemical property in other flaviviruses (Table 1). Other members of the JEV serocomplex possess amino acids with an aromatic ring side chain at this position, i.e., the E-F202 residue in JEV and the E-Y201 residue in St. Louis encephalitis and Murrey Valley encephalitis virus (MVEV). This residue is also conserved in other encephalitic flaviviruses, including the ZIKV E-L201 and tick-borne encephalitic virus E-V198. The equivalent location of the E protein is occupied by the hydrophobic methionine in all four serotypes of DENV. In agreement with the structural and functional importance of the WNV E-Y201 residue, the equivalent DENV-2 E-M196 residue is crucial for the membrane fusion process^26^. It is likely that a hydrophobic residue at the position equivalent to the WNV E-Y201 residue is crucial for the structure–function of most, if not all flavivirus E proteins. This is of significance for the rational design of candidate LAVs as previously reported mutations in the EDI-EDII hinge region did not support the development of a flavivirus-common attenuation process^40–42^. For example, the E-T198F mutation neighboring the E-Y201 residue can partially attenuate the neuroinvasive phenotype of WNV in 5-week-old C57BL6/J mice challenged via the subcutaneous route with 100 pfu, and the equivalent E-F193 residue is also important for the structure–function of the DENV-1 and DENV-2 E protein^26,31,41^. But the equivlent ZIKV E-F198T mutation resulted in no demonstrable difference^41^. The E-S277I mutation was previously shown to fully attenuate the neuroinvasive phenotype of MVEV in Swiss Webster mice^42^. While the MVEV E-S277I mutant exhibits the loss of HA activity indicative of interference with the membrane fusion process, the MVEV E-S277 residue is part of a Ser–Ser–Ser/Asn–Thr consensus sequence in the fourth motif of the EDI-EDII hinge region of flaviviruses in the JEV serocomplex, further limiting the application to the development of a flavivirus-common attenuation strategy. While mutations of the E-L135 residue led to the attenuated phenotype of DENV-2 in mosquitoes, the recovery of multiple DENV E-L135 mutants and the WNV E-I135N mutant was not successful following transfection of Vero cells and all DENV-2 E-L135 mutants had to be rescued in C6/36 cells^31^. Because recombinant live-attenuated flavivirus vaccines are currently manufactured in Vero cells^43^, a World Health Organization-approved cell substrate, the application of mutations of the E-L135 residue in mosquito C6/36 cells to the rational design of candidate LAVs could be potentially difficult.

**Fig. 5:**
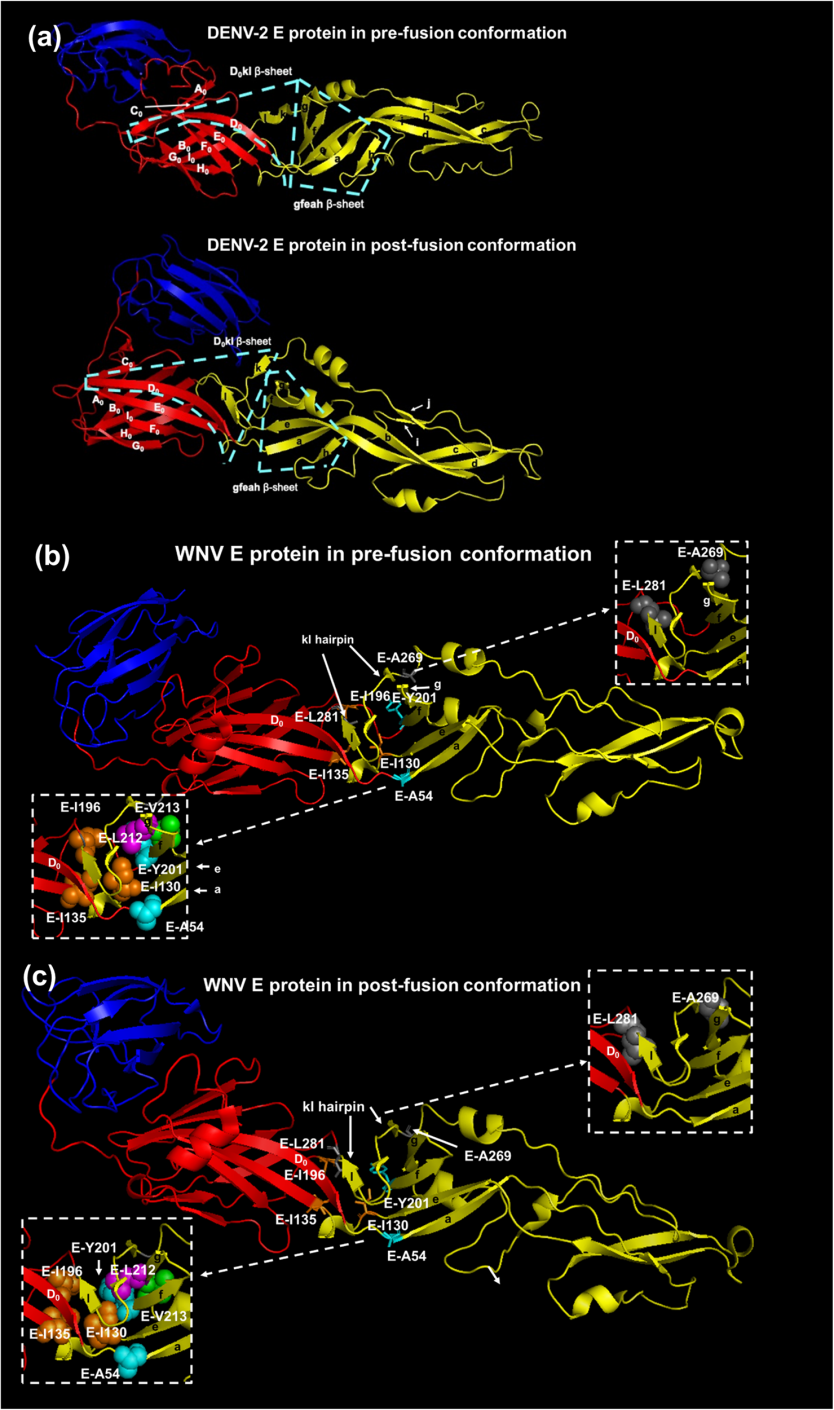
Six flavivirus-conserved hydrophobic residues crucial for the structure–function of the E protein. The two β-sheets at the EDI-EDII interface are highlighted in the pre-fusion and post-fusion conformations of DENV-2 E protein with cyan dash line in (**a**) because the crystal structure of WNV E protein is only available in pre-fusion conformation. The three domains of the E protein are colored in red (EDI), yellow (EDII), and blue (EDIII). The six flavivirus-conserved residues in the EDI-EDII hinge region of the pre-fusion (**b**) and post-fusion (**c**) conformations of WNV E protein are shown with stick (PDB ID:2HG0). The post-fusion conformation of WNV E protein is predicted using the post-fusion conformation of St. Louis encephalitis virus E protein (PDB ID: 4FG0). The detailed view highlighted the location of the gfeah and D_0_kl β-sheets. The E-A54 and E-Y201 residue at the N-terminus of the a and f β-sheets are highlighted with cyan spheres, respectively. The E-Y201 residue is proximal to the flavivirus-conserved E-L212 (green) and E-V213 (magenta) residues in the fg hairpin motif. The E-I130, E-135, and E-I196 residues are labeled with orange spheres. The E-A269S and the E-L281N mutation in the fourth motif of the EDI-EDII hinge region are shown in gray and are part of the klD_0_ β-sheet.

**Fig. 6:**
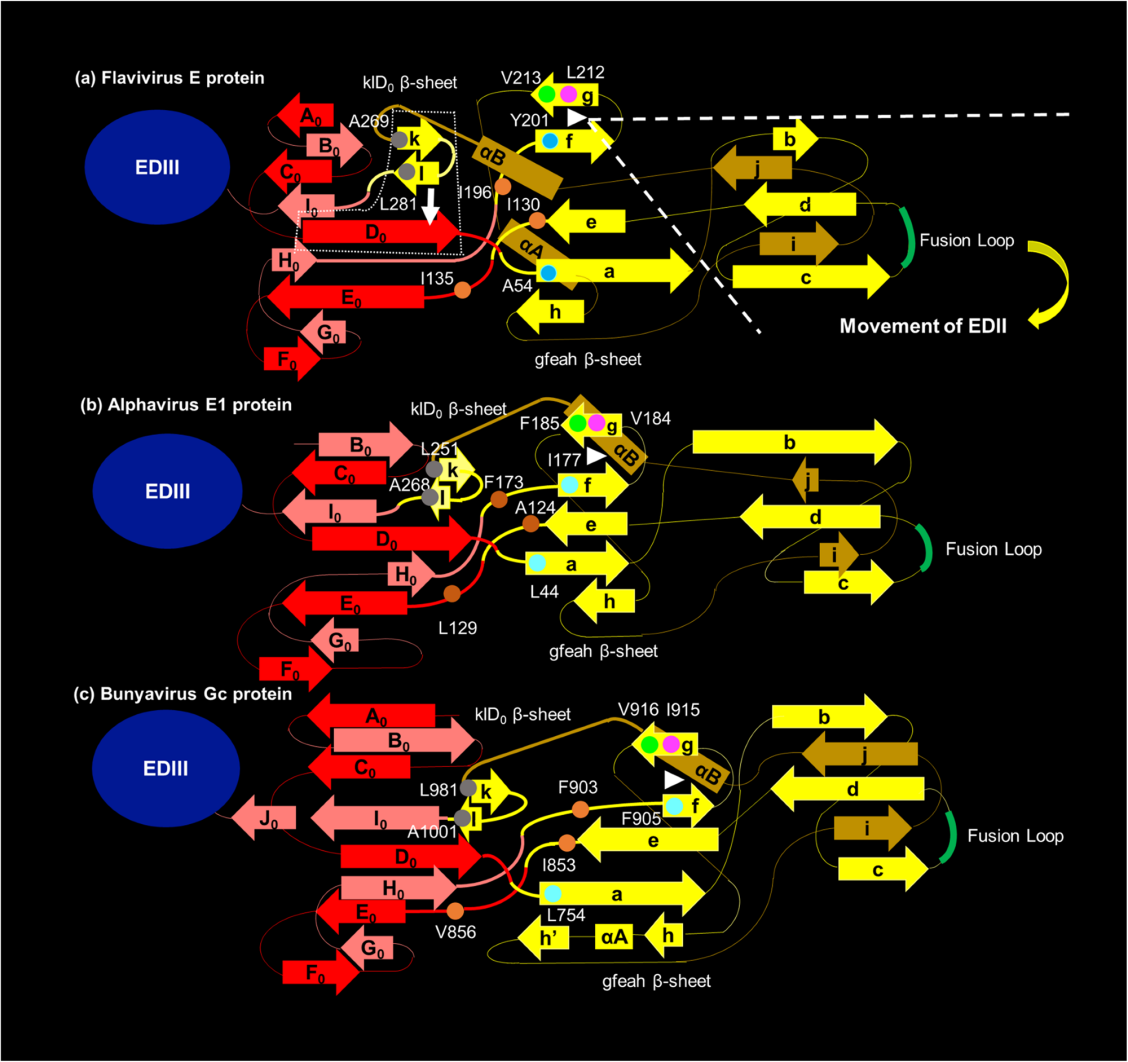
Proposed model for the arbovirus conserved hinge effect to control the EDI-EDII interdomain movement of E protein. **a** represents the proposed role of each flavivirus-conserved hydrophobic residue in exerting the hinge effect at the EDI-EDII interface of E protein. For simplicity, EDIII independent from the EDI-EDII interdomain movement is shown in a blue oval. The four motifs of the EDI-EDII hinge region are highlighted in thick lines. The WNV E-A54 and E-Y201 residues are at the base of the gfeah β-sheet, reflecting their importance in the relative movement of EDII in relation to EDI. The E-Y201 residue and the flavivirus-conserved E-L212 (magenta) and E-V213 (green) residues form the fg hairpin (white triangle). The gfeah β-sheet rotates along the fg hairpin to change the orientation of EDII (highlighted by the white dash line). The movement of EDII triggers the rotation of A_0_C_0_D_0_E_0_F_0_ β-sheet of the EDI. The new conformation of the D_0_ β-strand is stabilized by the hydrogen bonds with the l β-strand separated from the kl hairpin structure of EDII (indicated by the white arrow). The eqivalent hydrophobic residues in the alphavirus E1 proteins and bunyavirus Gc proteins are predicted using the structural alignment function of Modeller and Pymol software packages, reflecting that there is a putative common mechanism underlying the conformational change of the class II fusion proteins from dimer to trimer. The numbering is based on Semliki Forest virus (SFV) E1 protein (**b**) and Rift Valley fever virus (RVFV) Gc protein (**c**). The fg hairpin of alphavirus E1 protein and bunyavirus Gc protein (white triangle) both consist of three conserved hydrophobic amino acids. Specifically, the base of the fg hairpin motif at the EDI-EDII hinge region of SFV involves the alphavirus-conserved SFV E1-I177 (cyan), E1-V184 (margenta), and E1-F185 (green) hydrophobic residues. The fg hairpin motif is proximal to three alphavirus-conserved SFV E1-A124, E1-L129, and E1-F173 hydrophobic residues (shown in orange) equivalent to WNV E-I130, E-I135, and E-I196 residues, respectively. The base of the a β-strand of SFV E1 protein consists of the alphavirus-conserved E1-L44 residue (cyan), equivalent to the WNV E-A54 residue. And, the kl hairpin of the SFV E1 protein is potentially stabilized by two alphavirus-conserved hydrophobic amino acids, including the E1-L251 and E1-A268 residues (shown in gray), resembling the WNV E-A269 and E-L281 residues, respectively. Similarly, the fg hairpin motif at the EDI-EDII hinge region of RVFV Gc protein may constitute the bunyavirus-conserved RVFV Gc-F905 (cyan), Gc-I915 (margenta), and Gc-V916 (green) residues. The core of the RVFV Gc protein EDI-EDII hinge region also contains three bunyavirus-conserved hydrophobic amino acids, including the RVFV Gc-W855, Gc-L859, and Gc-F903 residues (show in orange), which resemble the relative location of the WNV E-I130, E-I135, and E-I196 residues, respectively. The close proximity between flavivirus-conserved WNV E-A54 and I130 residues is followed by the analogous RVFV Gc-L754 (cyan) and Gc-W855 (orange) residues, respectively. The kl hairpin of the RVFV Gc protein is potentially stabilized by the Gc-L981 and Gc-A1001 residues (shown in gray) conserved across different bunyaviruses.

In terms of the mechanism of the altered phentoypes, each of the seven amino acids occupies a key location between the gfeah and klD_0_ β-sheets at the EDI-EDII interface (Fig. 5a)^21^. The gfeah β-sheet rotates along the axle of fg hairpin to change the orientation of EDII and expose the fusion loop (FL) (Fig. 5b)^23^. The rotation of the gfeah β-sheet triggers the rearrangement of the three β-strands in the klD_0_ β-sheet to form hydrogen bonds between l and D_0_ β-strands in the E protein trimer^20^. The locations reveal the importance of the seven flavivirus-conserved hydrophobic amino acids in the EDI-EDII hinge region in the conformational change of the E protein from dimer to trimer (Fig. 6a). The WNV E-Y201 residue in the f β-strand is proximal to the two flavivirus-conserved hydrophobic amino acids in the g β-strand, e.g., the WNV E-L212 and E-V213 residues, and mediate the hydrophobic interaction forming the fg hairpin structure key to the EDI-EDII interdomain movement^44^. Disruption of the fg hairpin structure by the E-Y201P mutation is likely compensated by the consensus substitution of the proline residue with leucine, following two serial passages in Vero cells. Therefore, the stabilization of the E-Y201P mutation will require the change of all three nucleotides to the CCA codon that reflects the codon bias of WNV instead of the change of the two nucleotides in this study^45^. Alternatively, the emergence of the SNV encoding the E-M240V mutation in the P0 and P1 stocks may also be due to the E-Y201P mutation in the consensus sequence. The E-M240 residue is part of the ij loop that mediates the contact between two E protein monomers and possibly compensates for the dissociation of the homodimer prior to the formation of the E protein trimer^18,46^. In the same gfeah β-sheet, the 8.5 Å distance between the E-A54 residue and the E-I130 residue is within the distance for the hydrophobic interaction between amino acids and provides an explanation for the attenuating effect of WNV E-A54S, E-A54I, and E-A54E mutations and the lethal phenotype of WNV E-I130N mutant^47^. Mutations of the E-A54 residue are tolerated as the hydrogen bonds between the a β-strand and other neighboring β-strands may maintain the proper folding of the gfeah β-sheet^28,48^. In contrast, the E-I130 residue at the center of the EDI-EDII hinge region is predicted to mediate hydrophobic interactions with the E-I135 and E-I196 residues in the second and third motifs of the EDI-EDII hinge region^47,49^. Mutations of the E-I130, E-I135, and E-I196 residues are detrimental for the structure–function of flavivirus E protein, as demonstrated by the lethal phenotype caused by amino acid substitutions at either of the three residues in DENV-2 and WNV^31^. Finally, the emergence of the E-I270L compensatory mutation associated with the E-A269S consensus mutation and the reversion of the E-L281N mutation likely reflect the role of the kl hairpin controlling the rearrangement of the klD_0_ β-sheet^20^. The k and l β-strands that contain the E-A269 and E-L281 residues are separated in the E protein trimer, respectively. The WNV E-A269 residue is the strictly conserved Gly–Ala sequence in the N-terminus of the fourth motif of the EDI-EDII hinge region between mosquito-borne flaviviruses. Similarly, all flaviviruses except for DENV-4 contain a leucine or isoleucine residue at the position equivalent to the WNV E-L281 residue, which has the presumed importance in controlling the shift of the l β-strand towards the D_0_ β-strand of EDI. It is important to note that the role of the gfeah β -sheet structure in governing the EDI-EDII interdomain movement is conserved between class II fusion proteins with corresponding residues that can be identified in alphavirus E1 protein and bunyavirus Gc protein (Fig. 6b, c). These residues can be the targets for the engineering of mutations that interfere with the membrane fusion process to develop a common attenuation process for arboviruses in different genera^50–52^.

Overall, we have evaluated an alternative approach to the attenuation of flaviviruses based on mutations in the EDI-EDII hinge region important for the conformational change of E protein from dimer to trimer, and identified potential hydrophobic residues to be targeted for rational design for live-attenuated flavivirus vaccine development, especially as these residues are conserved in many members of the flavivirus genus.

## Methods

### Cell lines

Three cell lines were used in this study. African green monkey Vero cells and baby hamster kidney (BHK)-21 cells were maintained in minimum essential media-α supplemented with 10% fetal bovine serum (FBS), 100 U/mL penicillin, 100 μg/mL streptomycin, 2 mM l-glutamine, and MEM vitamin solution at 37 °C with 5% CO_2_. *Aedes albopictus* C6/36 cells were propagated in media containing an equal volume of high-glucose Dulbecco’s Modified Eagle Medium, and Mitsuhashi and Maramorosch insect media supplemented with 10% FBS 100 U/mL penicillin, 100 μg/mL streptomycin, and 2 mM l-glutamine at 28 °C.

### Design and production of the mutant viruses

The cDNA infectious clone of WNV NY99 strain was used to engineer mutations by site-directed mutagenesis^32^. The two-plasmid system encodes the genomic region between the 5’ untranslated region (UTR) and the *NgoMIV* site of the NS1 gene in the 5’ plasmid and the genomic region between the *NgoMIV* site of the NS1 gene and the 3’ UTR in the 3’ plasmid. As summarized in Table 3, PCR site-directed mutagenesis was undertaken to introduce substitutions of the eight conserved hydrophobic residues in the EDI-EDII hinge region using the InFusion cloning method (Takara). Briefly, the E-A54 and E-A269 residues (hydrophobic index: 41) were changed to serine (hydrophobic index: −5) by the G1126U and G1771U nucleotide substitutions, respectively. The E-A54 residue was also replaced with isoleucine and glutamate by introducing the C1127A and G1126A + C1127U nucleotide changes, respectively. The E-I130, E-I135, E-I196 residues (hydrophobic index: 99), and E-L281 residue (hydrophobic index: 97) were replaced with asparagine (hydrophobic index: −28). The E-I130N, E-I135N, and E-I196N mutations are achieved by a single U→A nucleotide substitutions at genome positions 1355, 1370, and 1553, respectively. The E-L281N mutation required three nucleotode changes in the codon, including U1807A, U1808A, and G1809C. The E-V272 residue (hydrophobic index: 76) was mutated to threonine (hydrophobic index: 13) by two nucleotide substitutions at the first and second positions in the codon, G1780A and U1781C. Because there is no amino acid that contains an aromatic ring in the side chain and has lower hydrophobicity than tyrosine, the E-Y201 residue (hydrophobic index: 63) was changed to proline, which maintains the ring structure in the side chain and exhibits lower hydrophilicity at physiological pH (hydropohic index −46)^53,54^. The E-Y201P mutation involved the change of the first and second positions in the codon, including U1567C and A1568C. The engineered nucleotide substitutions in each plasmid were confirmed by Sanger sequencing. All plasmids were prepared using 500 ml of overnight XL1-Blue strain of *Escherichia coli* competent cells (Stratagene) cultures in 2× YT broth supplemented with ampicillin at 50 μg/mL and purified using the alkaline lysis method (Nucleobond® Xtra Maxi kit, Takara).

**Table 3:** Mutations of the eight flavivirus-conserved amino acids in the WNV EDI-EDII hinge region.

Location	Amino acid substitutions in the E protein	Nucleotide substitutions in the genome position		
		1st codon	2nd codon	3rd codon
Motif #1	A54S	G1126T		
Motif #2	I130N		T1355A	
	I135N		T1370A	
Motif #3	V196T		T1553A	
	Y201P	T1567C	A1568C	
Motif #4	A269S	G1771T		
	V272T	G1870A	T1871C	
	L281N	T1807A	T1808A	G1809C

Each of the eight flavivirus-conserved hydrophobic residues in the WNV EDI-EDII hinge region was replaced with an amino acid of lower hydrophobicity.

Ten μg of 5’ plasmid was digested with *AscI* and *NgoMIV*, followed by the treatment with calf intestinal alkaline phosphatase (CIP). Approximately 30 μg of 3’ plasmid was first digested with *MluI-HF* and treated with CIP. The digested 5’ and 3’ plasmids were purified using the Nucleospin® column. The purified 3’ plasmid was subsequently digested with *NgoMIV*, followed by an additional round of purification. The digested 5’ and 3’ plasmids were ligated with T4 DNA ligase at room temperature for two hours, followed by the inactivation at 70 °C for 15 min. The ligated fragment was digested with *XbaI* and protease K, followed by purification using the phenol-chloroform extraction and ethanol precipitation method. The viral genome was produced by in vitro transcription using the Ampliscribe T7 High Yield Transcription kit (Lucigen) at 37 °C for an hour.

Transfection of RNA transcript was conducted by electroporation method^14^. Briefly, 10 μl of RNA transcripts were transfected into four million Vero cells suspended in 400 μl of ice-cold Dulbecco’s phosphate buffer saline without Mg^2+^/Ca^2+^ using the BioRad GenePulser^®^ system with one pulse of single wave condition at 225 volts for 25 msec in a 4-mm cuvette. Electroporated cells were allowed to recover on ice for 15 min, seeded in a T75 flask with prewarmed media, and harvested at 4 days after transfection, when cytopathic effects became apparent in 70–80% of cells. All in vitro and in vivo experiments were performed using virus stocks harvested from transfected Vero cells without additional passage, except for sequence analysis of genetic stability of the viruses.

### Nucleotide sequencing analysis

The retention of engineered mutations by viable WNV mutants was confirmed by Sanger sequencing and next-generation sequencing (NGS) methods. Briefly, total RNA was extracted from 250 μl of virus stock using Direct-zol RNA Miniprep Plus kit (Zymo Research). Sanger sequencing was undertaken to determine the consensus sequence of each WNV mutant for up to five passages (P5) in Vero cells. Three overlapping fragments corresponding to part of the prM and E genes were amplified by one-step reverse transcription-polymerase chain reaction (RT-PCR) and purified from an agarose gel prior to Sanger sequencing^55^. Further, NGS was undertaken to investigate RNA population of the unpassaged (P0) or passage one (P1) stocks of WNV NY99ic strain and the respective mutants rescued in two independent replicates. The NGS libraries were constructed using random hexamer primers and sequenced on the NextSeq550 platform using a paired-end 75nt base protocol. The adapter sequence and reads below quality score of 30 were removed by Trimmomatic. The reads from each virus were downsampled to a mean coverage of ~3500 reads and aligned against the WNV NY99ic reference sequence to determine the consensus sequence, using Bowtie 2 v 2.4.1. All reads are sorted using SAMTools v 1.6 and PCR duplicates were removed using Picard Tools v 2.25.7. Single-nucleotide variants (SNVs) that exceed 1% frequency were identified, using Lofreq v 2.1.3.1. Shannon Entropy of each nucleotide position was determined, using the formula ${S}_{n}=-\frac{{\sum }_{i=1}^{n}{fi}\left({lnfi}\right)}{N}\,$, where *n* is the number of possible nucleotide identified, *fi* is the observed frequency of a variant, and *N* is the total number of clones analyzed^4,40^.

### Infectivity of WNV mutants

Two methods were used to determine virus infectivity; plaque assays and 50% tissue culture infectious dose (TCID_50_). Plaque assays were performed in Vero cells to determine the infectivity of P0 stocks and P1 stocks and the data derived from the P1 stocks were used to determine the genome copy-to-plaque-forming unit (pfu) ratios. Fifty μl of varying tenfold serial dilutions of the virus was inoculated into 24-well plates, adsorbed at 37 °C for 60 min, and maintained in culture media supplemented with 1.5% carboxymethylcellulose at 37 °C with 5% CO_2_ for 5 days. Plaques were fixed with 10% formol saline and stained with 1% crystal violet solution. The multiplication kinetics of WNV mutants in Vero and BHK-21 cells was determined by TCID_50_. One hundred μl of culture supernatant collected from the respective cell lines was inoculated into 96-well microtiter plates in duplicate and tenfold serially diluted, followed by the addition of 100 μl Vero cells (2 × 10^6^ cells/ml) into each well. Cultures of infected Vero cells were stained with 0.5% amido black solution containing 35% of isopropyl alcohol and 10% of glacial acetic acid at 7 days post infection (dpi). Titers were calculated using the Muench-Reed method.

### Multiplication curve of WNV mutants

The multiplication kinetics of each mutant were compared to WNV NY99ic in Vero and BHK-21 cell lines. Virus stock was added to 24-well plates in triplicate at the multiplicity of infection (MOI) of 0.1 and adsorbed at 37 °C for 60 min, followed by the removal of inoculum and two washes with DPBS prior to the addition of 1 ml of fresh culture media into each well. The multiplication kinetics of each mutant in Vero and BHK-21 cells was determined using triplicate samples collected every 24 h up to 7 dpi and titrated by TCID_50_ assay as described above.

### Analysis of genome-pfu ratios of WNV mutants

The genome-to-pfu ratios of each virus were determined using samples harvested after infection of mutant viruses once in Vero cells. Culture supernatant harvested from transfected cells was inoculated into 24-well plates in duplicate at MOI of 0.1, adsorbed at 37 °C for 60 min, washed twice with DPBS, and overlayed with 1 ml of fresh culture media. Samples were aliquoted at 3 dpi and stored at −80 °C. One aliquot of each sample was titrated by plaque assay. Total RNA was extracted from a separate aliquot using the Direct-zol RNA miniprep plus columns (Zymo Research). Viral RNA in each sample was analyzed by quantitative RT-PCR using the pan-flavivirus mFU1 and CFD2 primer set and a WNV-specific probe conjugated with 6-carboxyfluorescein at the 5’ terminus (5’-TGCGTGAAGTTGGCACCCGGCCT-3’)^56^. Briefly, 2 μl of RNA extract was amplified in a 20 μl reaction using the following cycling parameters: reverse transcription at 50 °C for 30 min, hot-start activation at 95 °C for 15 min, and 45 cycles of denaturation at 95 °C for 15 s and annealing and amplification at 48 °C for 3 min with continuous fluorescence detection. The quantity of viral RNA in each sample was calculated using the standard curve generated from serially diluted full-length RNA transcripts derived from the infectious clone.

### Serial passage of WNV mutants

Triplicate wells of Vero cells were infected with each mutant or WNV NY99ic at MOI of 0.1, maintained at 37 °C with 5% CO_2_ and harvested at 3 dpi, which corresponds to the plateau phase in the multiplication kinetics of WNV NY99ic. Two aliquots were collected from each well for titration of infectivity using plaque assay and infection of Vero cells for a total of five passages.

### Hemagglutination activity of WNV

Stocks of each mutant prepared in C6/36 cells infected at MOI of 0.001 were used for hemagglutination (HA) assay. Gander erythrocytes (Innovative Research) was used for all HA assay, following the protocol as previously described^57^. Each mutant and WNV NY99ic were serially diluted in twofold steps, and the HA activity was determined at pHs between pH 5.8 and pH 7.4 in 0.2 pH increments. HA titers were calculated based on the reciprocal of the highest dilution leading to the complete agglutination. The optimal pH for the HA activity of each mutant was determined by the pH yielding the highest HA titer.

### Neuroinvasive phenotype of WNV mutants in mice

Groups of 4-week-old Swiss Webster mice (Charles River) were used to investigate the neuroinvasive phenotype of WNV mutants. Five male and five female mice were inoculated with 500 pfu of a particular WNV mutant or the wild-type (wt) WNV NY99ic via the intraperitoneal (i.p.) route and monitored for 14 days. Animals were euthanized after 20% or greater weight loss or the onset of neurotropic disease. All animals were euthanized by inhalation of carbon dioxide, with displacement of 30–70% of euthanasia chamber volume per minute. The brain of each mouse was collected after euthanasia for the quantification of tissue viral load using qRT-PCR as described above. Ct value above 33 was considered negative detection as previously described^58^. Serum samples were collected from mice euthanized at the endpoint of the study to determine serum-neutralizing activity by 50% plaque reduction neutralization test (PRNT_50_)^59^. Briefly, heat-inactivated serum samples were serially diluted in twofold steps, mixed with ~100 pfu of WNV NY99 strain, incubated at 37 °C for 60 min, and inoculated into 24-well plates. Plaques were stained at 5 dpi as described above. PRNT_50_ titer was defined by the reciprocal of the highest dilution that led to 50% reduction in plaque number compared to the virus control. All procedures were in compliance with the National Institutes of Health guide for the care and use of laboratory animals and approved by the Institutional Animal Care and Use Committee, Kansas State University.

### Statistical analysis

Infectivity, genome-pfu ratio, and mouse brain viral load of WNV mutants were compared against WNV NY99ic using one-way analysis of variance (ANOVA). Multiplication kinetics of WNV mutants in Vero and BHK-21 cells and infectivity of serially passaged WNV mutants were analyzed using two-way ANOVA. A pairwise comparison between each mutant and the parental wt WNV NY99ic control was performed using Dunnett’s test. Survival outcomes of mice challenged with WNV mutants were compared using the Kaplan–Meier survival curve. All statistical analysis was performed using the Prism 9 program (GraphPad Software).

### Reporting summary

Further information on research design is available in the Nature Research Reporting Summary linked to this article.

### Supplementary information

Supplementary tables

Reporting Summary

## Data Availability

All unique biological materials and the corresponding datasets generated and analyzed during the current study are available from the corresponding author on reasonable request.
