# Supplementary material for: Identification of the flavivirus conserved residues in the envelope protein hinge region for the rational design of a candidate West Nile live-attenuated vaccine
Source: NPJ Vaccines. 2023 Nov 6;8:172. doi: 10.1038/s41541-023-00765-0 (PMC10628263; doi:10.1038/s41541-023-00765-0)
Supplement: Supplementary file 1 — Supplementary tables [file 41541_2023_765_MOESM1_ESM.pdf]

Supplementary Table 1 Summary of SNVs >1% identified in the P0 stocks of WNV NY99ic and the respective mutants.

Virus	Replicate	Genomic Position	Major Base	Variant Base	Genome Region	Residue Change	Frequency (%)
WNV NY99ic	1	5166	G	A	NS3	-	2.1
		10888	U	A	3'UTR	-	1.3
	2	414	A	C	Capsid	-	1.5
		491	A	U	prM	K9M	23.7
		546	A	G	prM	-	34.6
		808	A	G	prM	T115A	3.1
		2421	U	G	E	F485L	1.0
		3061	C	G	NS1	L198V	7.3
		3786	A	C	NS2A	-	5.1
		5166	G	A	NS3	-	2.9
		6203	G	A	NS3	R531K	1.0
		8738	A	U	NS5	Q353L	11.8
		9072	C	U	NS5	-	4.5
		10888	U	A	3'UTR	-	1.7
E-A54S	1	5166	G	A	NS3	-	1.9
		8077	U	C	NS5	F133L	13.6
	2	2732	U	C	NS1	V88A	1.3
		3612	A	G	NS2A	-	1.1
		4483	A	G	NS2B	N89D	25.2
		4690	A	U	NS3	T27S	1.1
		5166	G	A	NS3	-	2.5
		5997	C	U	NS3	-	5.2
E-Y201P	1	546	A	G	prM	-	1.2
		866	A	U	prM	Y134F	1.2
		1920	A	U	E	-	1.1
		1968	A	G	E	-	1.3
		2313	A	C	E	-	2.9
		3477	A	G	NS1	-	1.9
		4920	C	U	NS3	-	2.6
		4950	A	C	NS3	-	1.5
		5166	G	A	NS3	-	1.4
		5369	A	G	NS3	N253T	1.7
		5917	A	G	NS3	T436A	1.3
		6203	G	A	NS3	R531K	1.1
		6634	U	C	NS4A	-	1.1
		8352	A	C	NS5	-	1.1
		10370	C	U	NS5	T897I	2.5
		10421	A	C	3'UTR	-	2.9
		10913	A	C	3'UTR	-	2.6
	2	831	A	G	prM	-	2.9

		1684	A	G	E	M240V	1.3
		2679	A	G	NS1	-	1.2
		2745	A	G	NS1	-	3.5
		3205	A	G	NS1	T246A	3.2
		4641	A	G	NS3	-	4.0
		5166	G	A	NS3	-	1.7
		5812	G	A	NS3	D401N	1.3
		6117	A	U	NS3	-	1.1
		6203	G	A	NS3	R531K	1.3
		7662	A	G	NS4B	-	3.2
		7777	A	U	NS5	I33F	1.2
		7794	A	C	NS5	-	1.2
		8361	C	A	NS5	-	1.6
		8889	A	G	NS5	-	1.4
		10769	A	G	3’UTR	-	3.3
E-A269S	1	1774	A	C	E	I270L	9.9
		2982	C	U	NS1	-	9.5
		5166	G	A	NS3	-	2.0
		9126	A	G	NS5	-	1.0
	2	406	A	G	Capsid	K104E	3.2
		683	C	U	prM	A73V	1.7
		710	C	A	prM	T82N	1.8
		1162	A	G	E	S66G	2.3
		2234	A	G	E	D423G	2.7
		2313	A	C	E	-	2.2
		2679	A	G	NS1	-	1.1
		4396	A	C	NS2B	I60L	3.2
		4614	A	C	NS3	-	1.5
		4965	A	G	NS3	-	1.9
		5166	G	A	NS3	-	2.2
		5250	A	G	NS3	I213M	1.4
		5346	A	U	NS3	-	1.2
		5571	A	C	NS3	-	3.4
		6912	A	G	2K	-	1.7
		6996	C	U	NS4B	-	5.5
		7707	A	G	NS5	-	2.4
		8523	C	A	NS5	N281K	1.4
		9637	U	A	NS5	F653I	2.0
		10306	A	C	NS5	-	2.7
		10408	C	U	3’UTR	-	2.1
		10650	A	G	3’UTR	-	1.5
		10740	A	C	3’UTR	-	1.0
		10750	C	U	3’UTR	-	1.4
		10778	A	G	3’UTR	-	4.4
E-V272T	1	217	A	C	Capsid	K41Q	14.3

		1907	C	U	E	T314I	1.6
		2052	U	C	E	-	2.3
		2581	A	G	NS1	T38A	14.4
		4238	U	C	NS2B	V7A	3.7
		5166	G	A	NS3	-	2.1
		7626	U	C	NS4B	-	2.0
		10888	U	A	3'UTR	-	1.1
	2	106	A	G	Capsid	K4E	1.4
		287	C	A	Capsid	A64E	3.9
		913	A	C	prM	M150L	5.3
		1311	A	C	E	-	1.9
		1800	U	C	E	-	2.3
		2427	A	C	E	-	1.8
		3593	C	G	NS2A	L24V	4.6
		3677	A	G	NS2A	Y51C	6.0
		4497	A	C	NS2B	-	5.4
		5166	G	A	NS3	-	1.8
		6263	U	C	NS3	V551A	1.1
		7420	A	C	NS4B	M169L	14.0
		8164	A	G	NS5	I162V	3.4
		9628	A	U	NS5	T650S	1.0
		9660	C	A	NS5	-	1.5
		9861	A	G	NS5	-	2.8
		10350	A	C	NS5	-	3.3
		10888	U	A	3'UTR	-	1.8
E-L281N	1	4292	A	C	NS2B	D25A	1.2
		4481	U	C	NS2B	M88T	5.4
		4719	A	U	NS3	-	15.1
		5166	G	A	NS3	-	2.0
		5325	A	G	NS3	-	13.0
		8457	C	U	NS5	-	1.4
		8769	G	A	NS5	-	7.0
		10888	U	A	3'UTR	-	1.5
	2	2222	C	U	E	A419V	2.3
		3961	A	C	NS2A	I146L	34.7
		3964	A	C	NS2A	T147P	6.4
		5166	G	A	NS3	-	1.9
		5938	A	G	NS3	I443V	2.0
		6646	U	C	NS4A	-	1.0
		9724	U	G	NS5	S682A	5.5
		10888	U	A	3'UTR	-	1.3

Supplementary Table 2 Summary of SNVs >1% identified in the P1 stock of WNV NY99ic and the respective mutants.

Virus	Repeat	Genomic Position	Major Base	Variant Base	Gene Region	Residue Change	Frequency (%)
WNV NY99ic	1	414	A	C	Capsid	-	1.6
		491	A	U	prM	K9M	28.7
		546	A	G	prM	-	38.9
		808	A	G	prM	T115A	5.2
		3061	C	G	NS1	L198N	6.9
		3786	A	C	NS2A	-	4.7
		5116	G	A	NS3	-	1.5
		6203	G	A	NS3	R531K	1.1
		8738	A	U	NS5	Q353L	3.1
		9072	C	U	NS5	-	4.5
	2	638	C	G	prM	A58G	1.4
		1274	A	C	E	N103T	2.8
		1708	A	U	E	T248S	1.1
		1863	A	G	E	-	1.4
		5166	G	A	NS3	-	1.7
		6203	G	A	NS3	R531K	1.4
		9584	A	G	NS5	N103T	2.1
E-A54S	1	5166	G	A	NS3	-	1.4
		6203	G	A	NS3	R531K	1.0
		8077	U	C	NS5	F133L	7.9
	2	4483	A	G	NS2B	N89D	21.0
		5166	G	A	NS3	-	1.6
		5997	C	U	NS3	-	5.1
E-Y201P	1	866	A	U	prM	Y134F	1.3
		1568	C	A	E	P201H	2.9
		1920	A	U	E	-	1.4
		2145	C	A	E	-	1.2
		2313	A	C	E	-	2.9
		3477	A	G	NS1	-	2.4
		4920	C	U	NS3	-	2.7
		4950	A	C	NS3	-	1.3
		5166	G	A	NS3	-	1.7
		5369	A	G	NS3	N253S	1.2
		6203	G	A	NS3	R531K	1.1
		6321	A	C	NS3	-	1.1
		8815	A	G	NS5	T379A	1.1
		10370	C	U	NS5	T897I	2.4
		10421	A	C	3’UTR	-	1.9
		10833	A	G	3’UTR	-	2.4

		10913	A	C	3’UTR	-	2.3
	2	831	A	G	prM	-	2.5
		1568	C	U	E	P201L	9.6
		1568	C	A	E	P201H	8.0
		1684	A	G	E	M240V	6.9
		2679	A	G	NS1	-	1.4
		2745	A	G	NS1	-	3.2
		2858	A	G	NS1	N130S	5.5
		2991	C	U	NS1	-	3.6
		3205	A	G	NS1	T246A	2.1
		4641	A	G	NS3	-	4.1
		5166	G	A	NS3	-	1.8
		5812	G	A	NS3	D401N	1.5
		7662	A	G	NS4B	-	5.4
		8356	U	A	NS5	S226T	1.4
		8361	C	A	NS5	-	1.3
		8889	A	G	NS5	-	2.0
		10769	A	G	3’UTR	-	2.3
E- A269S	1	1774	A	C	E	I270L	9.2
		2982	C	U	NS1	-	9.3
		5166	G	A	NS3	-	1.4
		6203	G	A	NS3	R531K	1.1
	2	406	A	G	Capsid	K104E	2.5
		683	C	U	prM	A73V	1.6
		1162	A	G	E	S66G	2.3
		1491	A	C	E	-	1.2
		2234	A	G	E	D423G	1.2
		2313	A	C	E	-	2.6
		2679	A	G	NS1	-	1.3
		4396	A	C	NS2B	I60L	3.5
		4614	A	C	NS3	-	1.1
		4965	A	G	NS3	-	3.5
		5166	G	A	NS3	-	1.4
		5250	A	G	NS3	I213M	1.3
		5346	A	U	NS3	-	1.1
		5571	A	C	NS3	-	3.8
		6203	G	A	NS3	R531K	1.5
		6912	A	G	2K	-	2.1
		6996	C	U	NS4B	-	5.0
		7707	A	G	SN5	-	1.8
		8523	C	A	NS5	N281K	1.8
		9637	U	A	NS5	F653I	2.9
		10306	A	C	NS5	-	2.7
		10369	A	C	NS5	T897P	1.4
		10408	C	U	3’UTR	-	1.6

		10650	A	G	3’UTR	-	1.3
		10740	A	C	3’UTR	-	1.1
		10778	A	G	3’UTR	-	5.7
E-V272T	1	217	A	C	Capsid	K41Q	11.0
		1907	C	U	E	T341I	1.7
		2052	U	C	E	-	2.3
		2581	A	G	NS1	T38A	10.0
		4238	U	C	NS2B	V7A	3.5
		5166	G	A	NS3	-	1.3
		6203	G	A	NS3	R531K	1.3
		7626	U	C	NS4B	-	2.3
	2	106	A	G	Capsid	K4E	1.9
		287	C	A	Capsid	A64E	4.2
		913	A	C	prM	M150L	5.8
		1311	A	C	E	-	2.2
		1800	U	C	E	-	3.2
		2427	A	C	E	-	1.5
		3595	C	G	NS2A	L24V	2.7
		3677	A	G	NS2A	Y51C	2.6
		4497	A	C	SN2B	-	4.1
		5166	G	A	NS3	-	1.6
		6203	G	A	NS3	R531K	1.2
		6263	U	C	NS3	V551A	1.6
		7420	A	C	NS4B	M169L	16.0
		8164	A	G	NS5	I162V	3.6
		9628	A	U	NS5	T650S	1.7
		9660	C	A	NS5	-	1.5
		9861	A	G	NS5	-	3.5
		10350	A	C	NS5	-	2.4
E-L281N	1	4481	U	C	NS2B	M88T	4.4
		4719	A	U	NS3	-	14.4
		5166	G	A	NS3	-	1.2
		5325	A	G	NS3	-	14.2
		6203	G	A	NS3	R531K	1.0
		8457	C	U	NS5	-	1.6
		8769	G	A	NS5	-	6.4
	2	2222	C	U	E	A419V	3.2
		3961	A	C	NS2A	I146L	36.5
		3964	A	C	NS2A	T147P	1.3
		5166	G	A	NS3	-	1.0
		5938	A	G	NS3	I443V	1.3
		6203	G	A	NS3	R531K	1.3
		6646	U	C	NS4A	-	1.4
		9724	U	G	NS5	S682A	1.3

Supplementary Table 3 Summary of SNVs >1% identified in the P0 stock of E-A54I and E-A54E mutants in comparison with WNV NY99ic control rescued simultaneously.

Virus	Repeat	Genomic Position	Reference Base	Variant Base	Gene Region	Amino Acid Change	Variant Frequency (%)
WNV NY99ic	1	417	A	G	C	-	5.0
		1527	A	C	E	-	7.6
		1955	C	T	E	T330I	1.2
		2502	A	G	NS1	-	17.8
		5166	G	A	NS3	-	2.3
		6165	T	C	NS3	-	3.3
		9195	A	T	NS5	-	5.3
		10178	C	T	NS5	T833I	1.9
		10524	C	A	3'UTR	-	1.6
		10888	T	A	3'UTR	-	1.9
	2	738	G	C	prM	-	1.9
		744	A	G	prM	-	2.3
		1561	A	T	E	N199Y	2.5
		2182	A	G	E	K406E	9.0
		2184	A	G	E	-	6.6
		4509	A	C	NS2B	-	4.7
		5166	G	A	NS3	-	2.7
		7403	A	G	NS4B	E163G	1.0
		8953	A	C	NS5	-	11.1
		9612	A	G	NS5	-	11.7
		10888	T	A	3'UTR	-	2.3
E-A54I	1	2618	A	C	NS1	H50P	18.6
		2847	A	T	NS1	-	4.7
		5166	G	A	NS3	-	2.5
		6462	A	G	NS3	-	1.9
		7341	G	A	NS4B	-	3.1
	2	5166	G	A	NS3	-	2.7
		8415	A	G	NS5	-	18.6
		10161	A	G	NS5	-	5.2
		10888	T	A	3'UTR	-	4.2
E-A54E	1	1336	A	C	E	K124Q	1.7
		1621	A	C	E	M219L	2.7
		1804	A	G	E	K280E	6.9
		2754	A	C	NS1	-	2.3
		5084	A	G	NS3	N158S	1.9
		5166	G	A	NS3	-	2.3
		6203	G	A	NS3	R531K	1.2
		7411	A	T	NS4B	T166S	3.6

	7461	T	C	NS4B	-	1.2
	7695	A	G	NS5	-	2.3
	8513	A	G	NS5	K278R	11.2
2	1403	C	G	E	P146R	1.0
	4009	C	T	NS2A	-	1.1
	5166	G	A	NS3	-	2.3
	5414	A	T	NS3	H268L	2.7
	5586	A	C	NS3	-	3.5
	5958	A	G	NS3	-	6.2
	6462	A	G	NS3	-	7.5
	6971	A	C	NS4B	Q19P	1.0
	9894	A	T	NS5	E738D	2.1

Supplementary Table 4 Summary of SNVs >1% identified in the P1 stock of E-A54I and E-A54E mutants.

Virus	Repeat	Genomic Position	Reference Base	Variant Base	Gene Region	Amino Acid Change	Variant Frequency (%)
E-A54I	1	2618	A	C	NS1	H50P	16.5
		2847	A	T	NS1	-	3.5
		5166	G	A	NS3	-	2.7
		6203	G	A	NS3	R531K	1.3
		6462	A	G	NS3	-	4.2
		7341	G	A	NS4B	-	3.9
		10888	T	A	3'UTR	-	1.3
	2	55	C	T	5'UTR	-	1.1
		5166	G	A	NS3	-	2.5
		6162	A	G	NS3	-	1.1
		8077	T	C	NS5	F133L	1.3
		8415	A	G	NS5	-	14.9
		8447	A	G	NS5	E256G	2.7
		8833	G	A	NS5	A385T	4.9
		10260	C	T	NS5	-	3.5
		10815	G	T	3'UTR	-	2.2
		10888	T	A	3'UTR	-	1.7
E-A54E	1	50	A	G	5'UTR	-	1.3
		1804	A	G	E	K280E	3.5
		1876	G	C	E	V304L	1
		1877	T	A	E	V304D	1
		2754	A	C	NS1	-	8.4
		5166	G	A	NS3	-	2.5
		7461	T	C	NS4B	-	13.3
		7695	A	G	NS5	-	6.7
		8513	A	G	NS5	K278R	16.9
		10852	C	T	3'UTR	-	8.1
		10888	T	A	3'UTR	-	2.6
	2	175	T	C	C	-	2.4
		272	T	C	C	I59T	2.3
		833	A	G	prM	K123R	1.2
		1558	A	G	E	T198A	1.8
		3288	A	G	NS1	-	7.9
		3871	A	T	NS2A	M116L	1.9
		5166	G	A	NS3	-	3.1
		5367	T	C	NS3	-	1.6
		5414	A	T	NS3	H268L	3.2
		5586	A	C	NS3	-	8.4
		5958	A	G	NS3	-	6.2
		6152	G	A	NS3	N498K	2.8

6165	T	C	NS3	N503H	6.8
6462	A	G	NS3	-	6.9
7777	A	C	NS5	N17T	1.1
7848	T	C	NS5	-	3
9894	A	T	NS5	E738D	1.2
10415	A	T	NS5	-	3.3
10888	T	A	3'UTR	-	2.7
