# Supplementary material for: Identification of the flavivirus conserved residues in the envelope protein hinge region for the rational design of a candidate West Nile live-attenuated vaccine
Source: NPJ Vaccines. 2023 Nov 6;8:172. doi: 10.1038/s41541-023-00765-0 (PMC10628263; doi:10.1038/s41541-023-00765-0)
Supplement: Supplementary file 2 — Reporting Summary [file 41541_2023_765_MOESM2_ESM.pdf]

nature portfolio

Corresponding author(s): Yan-Jang S. Huang
Last updated by author(s): 2023/07/14

Reporting Summary

Nature Portfolio wishes to improve the reproducibility of the work that we publish. This form provides structure for consistency and transparency in reporting. For further information on Nature Portfolio policies, see our Editorial Policies and the Editorial Policy Checklist.

Statistics

For all statistical analyses, confirm that the following items are present in the figure legend, table legend, main text, or Methods section.

n/a	Confirmed	
☐	☒	The exact sample size (n) for each experimental group/condition, given as a discrete number and unit of measurement
☐	☒	A statement on whether measurements were taken from distinct samples or whether the same sample was measured repeatedly
☐	☒	The statistical test(s) used AND whether they are one- or two-sided<br>*Only common tests should be described solely by name; describe more complex techniques in the Methods section.*
☒	☐	A description of all covariates tested
☒	☐	A description of any assumptions or corrections, such as tests of normality and adjustment for multiple comparisons
☐	☒	A full description of the statistical parameters including central tendency (e.g. means) or other basic estimates (e.g. regression coefficient) AND variation (e.g. standard deviation) or associated estimates of uncertainty (e.g. confidence intervals)
☒	☐	For null hypothesis testing, the test statistic (e.g. F, t, r) with confidence intervals, effect sizes, degrees of freedom and P value noted<br>*Give P values as exact values whenever suitable.*
☒	☐	For Bayesian analysis, information on the choice of priors and Markov chain Monte Carlo settings
☒	☐	For hierarchical and complex designs, identification of the appropriate level for tests and full reporting of outcomes
☒	☐	Estimates of effect sizes (e.g. Cohen's d, Pearson's r), indicating how they were calculated

Our web collection on statistics for biologists contains articles on many of the points above.

Software and code

Policy information about availability of computer code

Data collection	Software used for data collection is as follows:<br>1) Alignment of next-generation sequencing reads against the reference West Nile virus NY99 genome was performed with Bowtie2 v 2.4.1 and SAMTools v 1.6.<br>2) PCR duplicates were removed using Picard Tools v2.25.7.<br>3) Determination of depth overage and random downsampling of next-generation sequencing reads were performed using SAMTools v 1.6.<br>4) Shannon entropy of each mutant and control virus was calculated using R software package.
Data analysis	Software used for data analysis is as follows:<br>1) Infectivity, genome-plaque forming unit ratio, survival analysis, serum neutralizing activity, brain viral load, and Shannon entropy data were analyzed using GraphPad Prism.<br>2) All figures were generated using GraphPad Prism

For manuscripts utilizing custom algorithms or software that are central to the research but not yet described in published literature, software must be made available to editors and reviewers. We strongly encourage code deposition in a community repository (e.g. GitHub). See the Nature Portfolio guidelines for submitting code & software for further information.

Data

Policy information about availability of data

All manuscripts must include a data availability statement. This statement should provide the following information, where applicable:

- Accession codes, unique identifiers, or web links for publicly available datasets
- A description of any restrictions on data availability
- For clinical datasets or third party data, please ensure that the statement adheres to our policy

All unique biological materials and the corresponding datasets generated and analyzed during the current study are available from the corresponding author on reasonable request.

Research involving human participants, their data, or biological material

Policy information about studies with human participants or human data. See also policy information about sex, gender (identity/presentation), and sexual orientation and race, ethnicity and racism.

Reporting on sex and gender	Mouse challenge studies in this project were performed using equal number of male and female animals to rule out sex as a biological variable.
Reporting on race, ethnicity, or other socially relevant groupings	not applicable
Population characteristics	not applicable
Recruitment	not applicable
Ethics oversight	not applicable

Note that full information on the approval of the study protocol must also be provided in the manuscript.

Field-specific reporting

Please select the one below that is the best fit for your research. If you are not sure, read the appropriate sections before making your selection.

☒ Life sciences ☐ Behavioural & social sciences ☐ Ecological, evolutionary & environmental sciences

For a reference copy of the document with all sections, see nature.com/documents/nr-reporting-summary-flat.pdf

Life sciences study design

All studies must disclose on these points even when the disclosure is negative.

Sample size	No sample size calculation was performed. But the numbers of biological replicates and animals followed the standard acceptable to the field and previously published studies.<br>(1) All cell culture experiments were undertaken with three biological replicates.<br>(2) Groups of ten mice to investigate the neuroinvasive phenotype of West Nile virus mutants is the sample size implemented in previously published studies.<br>(3) Two biological replicates for the respective mutant at P0 and P1 passage is the sample size implemented in previously published studies.
Data exclusions	No data were excluded from analysis.
Replication	Rescue of mutants,
Randomization	not applicable
Blinding	not applicable

Reporting for specific materials, systems and methods

We require information from authors about some types of materials, experimental systems and methods used in many studies. Here, indicate whether each material, system or method listed is relevant to your study. If you are not sure if a list item applies to your research, read the appropriate section before selecting a response.

Materials & experimental systems

n/a	Involved in the study
☒	☐ Antibodies
☐	☒ Eukaryotic cell lines
☒	☐ Palaeontology and archaeology
☐	☒ Animals and other organisms
☒	☐ Clinical data
☒	☐ Dual use research of concern
☒	☐ Plants

Methods

n/a	Involved in the study
☒	☐ ChIP-seq
☒	☐ Flow cytometry
☒	☐ MRI-based neuroimaging

Eukaryotic cell lines

Policy information about cell lines and Sex and Gender in Research

Cell line source(s)	Vero cells, baby hamster kidney (BHK)-21 cells, and Aedes albopictus C6/36 cells used in this project were provided by the World Reference Center for Emerging Viruses and Arboviruses, University of Texas Medical Branch.
Authentication	We did not perform authentication during this project.
Mycoplasma contamination	We did not undertake mycoplasma testing during this project.
Commonly misidentified lines (See ICLAC register)	none

Animals and other research organisms

Policy information about studies involving animals; ARRIVE guidelines recommended for reporting animal research, and Sex and Gender in Research

Laboratory animals	Outbred Swiss Webster mice<br>Species Name: Mus musculus<br>Age: 3-4 weeks old<br>Source: Charles River Laboratories
Wild animals	Not applicable
Reporting on sex	Not applicable
Field-collected samples	Not applicable
Ethics oversight	Not applicable

Note that full information on the approval of the study protocol must also be provided in the manuscript.
